# Osteoporosis treatment using stem cell-derived exosomes: a systematic review and meta-analysis of preclinical studies

**DOI:** 10.1186/s13287-023-03317-4

**Published:** 2023-04-11

**Authors:** Xiaoyu He, Yangbin Wang, Zhihua Liu, Yiyong Weng, Shupeng Chen, Qunlong Pan, Yizhong Li, Hanshi Wang, Shu Lin, Haiming Yu

**Affiliations:** 1 Department of Orthopaedics, The Second Affiliated Hospital of Fujian Medical University, No. 950 Donghai Street, Quanzhou, 362000 Fujian Province China; 2 Centre of Neurological and Metabolic Research, The Second Affiliated Hospital of Fujian Medical University, No. 34 North Zhongshan Road, Quanzhou, 362000 Fujian Province China; 3 Group of Neuroendocrinology, Garvan Institute of Medical Research, 384 Victoria St, Sydney, Australia

**Keywords:** Exosome, Extracellular vesicle, Stem cell therapy, Osteoporosis, Bone loss, Meta-analysis

## Abstract

**Background:**

The increasing incidence of osteoporosis in recent years has aroused widespread public concern; however, existing effective treatments are limited. Therefore, new osteoporosis treatment methods, including stem cell transplantation and exosome therapy, have been proposed and are gaining momentum. Exosomes are considered to have greater potential for clinical application owing to their immunocompatibility. This study summarises the latest evidence demonstrating the efficacy of exosomes in improving bone loss in the treatment of osteoporosis.

**Main text:**

This systematic review and meta-analyses searched PubMed, Embase, and Cochrane Library databases from inception to 26 March 2022 for osteoporosis treatment studies using stem cell-derived exosomes. Six endpoints were selected to determine efficacy: bone mineral density, trabecular bone volume/tissue volume fraction, trabecular number, trabecular separation, trabecular thickness, and cortical thickness. The search generated 366 citations. Eventually, 11 articles that included 15 controlled preclinical trials and 242 experimental animals (rats and mice) were included in the meta-analysis.

**Conclusion:**

The results were relatively robust and reliable despite some publication biases, suggesting that exosome treatment increased bone mass, improved bone microarchitecture, and enhanced bone strength compared with placebo treatments. Moreover, stem cell-derived exosomes may favour anabolism over catabolism, shifting the dynamic balance towards bone regeneration.

**Supplementary Information:**

The online version contains supplementary material available at 10.1186/s13287-023-03317-4.

## Background

Osteoporosis is a disease characterised by reduced bone mass, microstructural destruction, and fragility fractures with a particularly high incidence in older adults, regardless of ethnicity [1–3]. It has become a serious global public health problem owing to ageing populations [4–6]. Fractures, particularly hip fractures and vertebral compression fractures (VCFs), are the most common and devastating osteoporotic complications. Those complications cause great suffering to patients and severely reduce their quality of life and increasing disability and mortality. The consequent disability and mortality impose a heavy burden on families worldwide and on the global society [2, 7, 8].

Existing osteoporosis treatments have various limitations. For example, although procedures such as vertebral augmentation can repair fractures and relieve local pain, they may be accompanied by increased risk of infection, cement extravasation, embolism, hematoma, and other negative effects [9]. Bisphosphonates are first-line osteoporosis medications that are typically taken for at least 3–5 years, but they have various undesirable side effects, including muscle pain and osteonecrosis of the jaw [10, 11]. Denosumab is a potent anti-absorptive medication that significantly increases bone mineral density (BMD). However, its association with a rebound increase in bone absorption, leading to a sharp decrease in BMD, and in turn increasing the risk of multiple vertebral fractures, contributed to its discontinuation [12].

Emerging novel methods for osteoporosis treatment such as stem cell transplantation and exosome therapy have recently garnered attention. Stem cells fall into two major categories: embryos and adult. Embryonic stem cells (ESCs), pluripotent stem cells, and mesenchymal stromal cells (MSCs), among multipotent cells, have been broadly used in the biomedical field. MSCs are usually preferred to ESCs because of their easy availability. However, exosomes may be more favourable than all stem cells as they are not immunogenic and have abundant sources [13].

Exosomes are nanoscale extracellular vesicles secreted by cells; these structures encapsulate biologically active substances, including microRNAs, lipids, and proteins [14]. Because major histocompatibility complex proteins are not expressed on the surface, exosomes can be used for transplantation therapy, which means exosome therapy rarely encounters rejection [13]. In addition, they have been reported to be involved in intercellular communication, various physiological and pathological processes, and play critical roles in angiogenesis, atherosclerosis, MSC repair, osteoclast activity, and osteoblast differentiation [13, 15, 16]. Liu et al. demonstrated in vitro and in vivo that exosomes transfer Fas to recipient MRL/lpr BMMSCs in order to reduce intracellular levels of miR-29b, thus improving MRL/lpr BMMSC function [17]. Yang et al. proved that MALAT1 in exosomes enhances osteoblast activity by mediating the miR-34c/SATB2 axis [18]. Carlos Castaño et al. revealed that exercise-induced exosomal miRNAs decrease hepatic FoxO1 expression, thus improving the glucose metabolism of hepatic cells [19].

In summary, exosomes carry cell-specific cargoes according to their parent cells and may promote functional recovery of cells and maintain the homeostasis of the internal environment to initiate repair and regeneration of bone through different signal transduction pathways [13, 20]. Therefore, exosomes can potentially be used to treat bone loss. Here, we performed meta-analyses to evaluate the efficacy of exosomes’ (derived from stem cells) capacity to ameliorate bone loss and osteoporosis.

## Materials and methods

### Systematic review

Systematic reviews and meta-analyses were interpreted and elaborated as per Preferred Reporting Items for Systematic Reviews and Meta-Analyses (PRISMA) and PRISMA 2020 [21, 22]. The registration number for this study is CRD42022337860.

### Search strategies

**Fig. 1:**
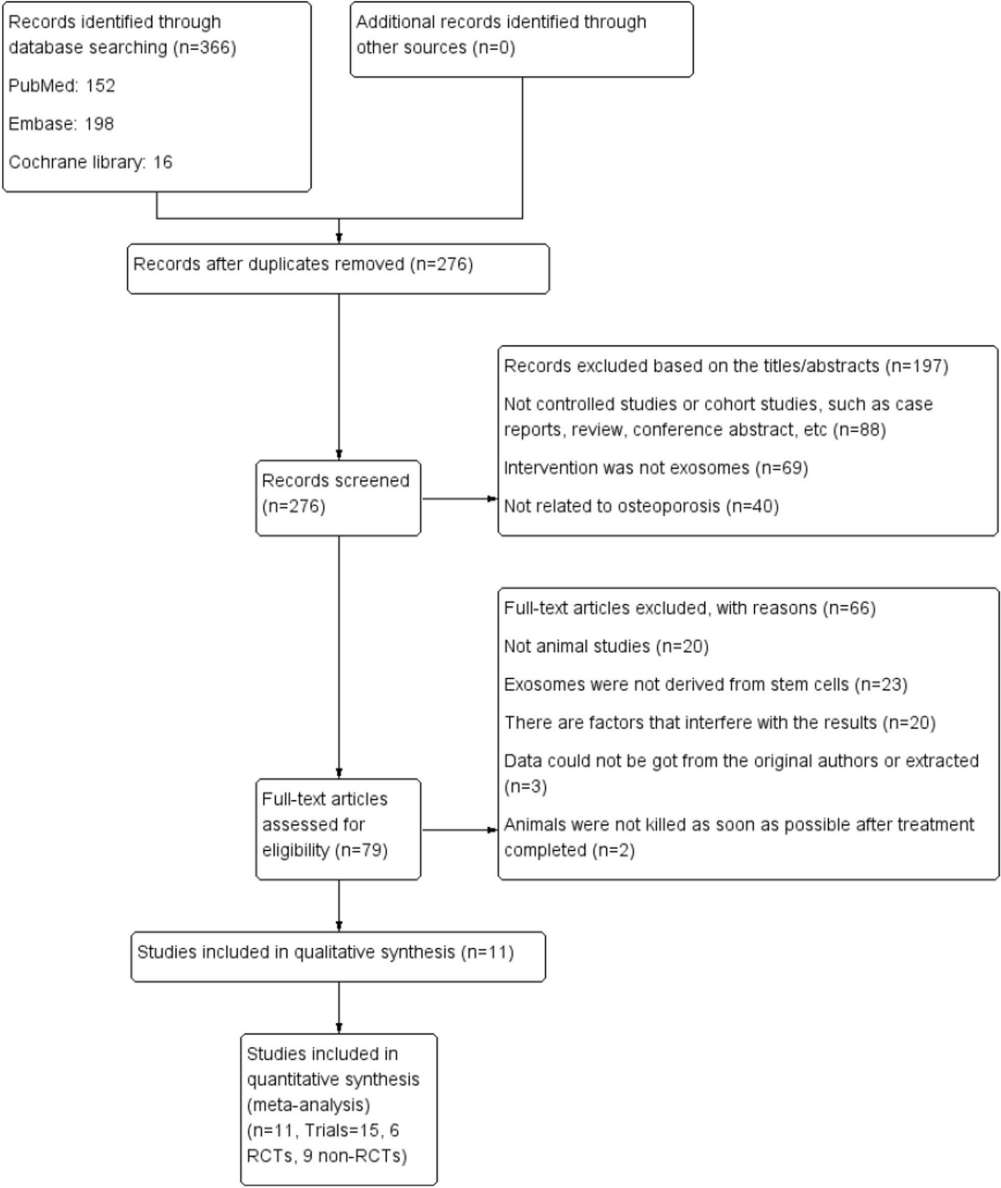
PRISMA flow diagram of literature search and selection of studies for meta-analysis

Two researchers (He Xiaoyu and Wang Yangbin) conducted independent and manual searches in PubMed, Cochrane Library, and Embase databases (from inception to 26 March 2022) using the Medical Subject Headings (MeSH) terms ‘extracellular vesicles’ or ‘exosomes’ and ‘osteoporosis’ (Fig. 1). The corresponding free words and Boolean operators (AND or OR) were important components of the literature search strategy (Additional file 1: Table S1). Duplicates were removed, and articles were subsequently selected according to the title and abstract in the first round of browsing. The second round of selection was based on reading of the full article in detail. The relevant data were then extracted according to the standards set in the study selection criteria, such as sample size, experimental subjects, study design, and intervention. During this process, our team resolved differences and disagreements through discussions. If uncertainty persisted, a third party made the decision (Yu Haiming and Lin Shu).

### Study selection criteria

#### Inclusion criteria

A. This review considered studies using animals, regardless of their species, sex, age, or disease models to explore the efficacy of exosome therapy with respect to bone repair.
B. Animals in one of the experimental groups were treated with exosomes derived from stem cells, whereas animals in the control group received only a placebo.
C. The study measured at least one of the following indicators: (1) BMD; (2) bone volume fraction (trabecular bone volume/total volume, Tb. BV/TV); (3) trabecular number (Tb. N); (4) trabecular thickness (Tb. Th); (5) trabecular separation/marrow thickness (Tb. Sp); and (6) cortical thickness (Ct. Th).
D. This study was a controlled trial.

#### Exclusion criteria

A. The study subjects were humans.
B. The data in the study could not be extracted or obtained from the original authors.
C. Data are incomplete or expressed as ratios or percentages.
D. The study was not a controlled trial, such as a case report, review, meeting, letter, survey, or satisfaction study.
E. The study was not published as a full text article in a journal.
F. All animals were treated using exosomes carrying a gene or other expression regulator which might interfere with the results of the outcome indicators.
G. Animals were not immediately killed after treatment, which could influence the outcome indicators.

### Required data extraction

Two researchers (He Xiaoyu and Wang Yangbin) extracted data from the retained studies. The data were then collated and checked by two other researchers (Liu Zhihua and Weng Yiyong); the controversial sections were discussed by the entire research team for resolution. If disagreements persisted, the matter was transferred to a third party (Yu Haiming and Lin Shu) to reach a consensus.

The main data extracted in this meta-analysis included the following six outcome assessment indices. (1) BMD is an important indicator for measuring bone mass and strength, which can reflect the degree of osteoporosis and assess fracture risk. (2) Tb. BV/TV reflects the bone mass of trabecular bone in different samples and is known as bone volume fraction (BVF). (3) Tb. N, Tb. Th, and Tb. Sp are main indices to evaluate bone trabecula spatial morphology and structure. (4) Ct. Th is a parameter reflecting the changes in cortical bone properties that contribute to a comprehensive understanding of bone growth and disease. All extracted data contained consecutive values with three decimals and the appropriate units. In addition, basic information and the detailed research design of the articles were collected. Basic information specifically included the first author, publication year, research type, modelling method, species, type, age, and sex. The details of the study designs included the source, sample size of experimental and control groups, route of administration, and frequency of application of stem cell-derived exosomes. The corresponding author was emailed for details when key research data and information were not mentioned in the paper.

### Quality assessment of included studies

The Systematic Review Centre for Laboratory Animal Experimentation’s (SYRCLE’s) risk of bias tool including sequence generation (selection bias), baseline characteristics (selection bias), allocation concealment (selection bias), random housing (performance bias), blinding (performance bias), random outcome assessment (detection bias), blinding (detection bias), incomplete outcome data (attrition bias), selective outcome reporting (reporting bias), and other sources of biases were used to assess the quality of included study trials [23]. Two researchers independently conducted the work.

### Statistical analysis

GetData Graph Digitizer, Review Manager (RevMan) 5.4.1, and Stata SE 15 were used for the systematic review and meta-analysis, data extraction, and processing operations, respectively. Mean and standard deviation (SD) data were collected. In studies presenting only the standard error of the mean (SEM), SEM was converted to SD according to the equation: SD = SEM × (√n) [24] (where ‘n’ refers to the animal number in the experimental or control group). Mean and SD were used as inputs in data processing tools to generate a weighted mean difference (WMD) and a standardised mean difference (SMD), as well as their respective 95% confidence intervals (95% CI). SMD was selected as the final effective indicator owing to the wide variations in the mean between studies and data using different units of measure.

Heterogeneity was evaluated using the Q statistic test and the I^2^ test. Heterogeneity between studies was considered when *P* ≤ 0.05 or *I*^2^ > 50%. If the judgement results of the two test methods for heterogeneity were contradictory, the I^2^ test result was taken as it is more reliable than the Q statistical test. Significant heterogeneity between studies was explained using subgroup analyses, sensitivity analyses, or other analyses. A fixed effect model was adopted based on the assumption that all studies were sampled from the same population; however, it was not employed for animal studies as this assumption could not be made. Therefore, a random-effects model was generally used.

Publication bias was assessed by constructing a funnel plot. The assumption was that the experimental data exceeded 10 sets; otherwise, the validity of the test was very low. Egger’s test was used to verify the authenticity of the asymmetry if the funnel plot showed slight asymmetry. *P* > 0.05 indicated that asymmetry did not exist. Meanwhile, the trim-and-fill method was used for data with a P value less than or equal to 0.05 to estimate the effect of publication bias on the results. In addition, a meta-based influence analysis was used as a sensitivity analysis to exclude the influence of a small sample size to determine the stability of the results. Finally, in the meta-analysis, results were considered significant when *P* < 0.05.

## Results

### Included studies

The PubMed, Embase, and Cochrane Library databases were searched using MeSH terms and free words to retrieve 152, 198, and 16 studies, respectively. Ninety duplicate studies were withdrawn, and 276 studies were retained for the next selection. Subsequently, 197 studies were removed based on their title and abstract. Finally, 11 studies (total of 15 trials involving 242 rats or mice) met the study selection criteria and were included in the meta-analysis after the full texts were read (Fig. 1) [25–35].

### Study characteristics

**Fig. 2:**
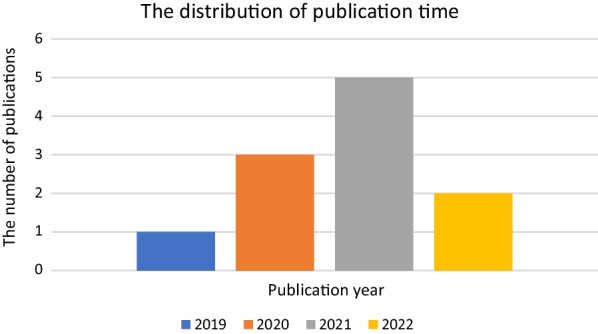
Publication year of the included studies

The 11 studies were from 2019, 2020, 2021, and 2022 (Fig. 2), and all 15 trials were conducted in China. The total sample size was 242 animals, among which 121 received exosome treatment and the remainder received a placebo. The characteristics of the experimental subjects (Table 1) and all 15 trials (Table 2), including (but not limited to) animal model used, sex, age, species, exosome type, and sampling area, were collected and listed. The exosomes used in the trials were derived from various stem cells from humans or rats/mice, including urine-derived stem cells (USCs) [25], ESCs [26], human umbilical cord MSCs (hucMSCs) [27], bone marrow-derived MSCs (BMSCs) [28–33], and adipose tissue-derived MSCs (ADSCs) [34, 35]. Although exosomes were derived from cells of different species, the results of each study included have independently demonstrated their effectiveness on osteoporosis in animal models. All trials directly compared the exosome-treated group with the placebo group. Drug delivery routes in trials primarily involved intravenous (IV) injection [25, 27–31, 33–35], although gavage [26] and periosteum injection were also used [32]. The frequency of exosome treatment was split between every other day, weekly, and twice a week. Only one trial did not mention the administration frequency [31]. Treatment cycles in all trials ranged from one week to six months. The collected outcomes involved BMD, Tb. BV/TV, Tb. N, Tb. Th, Tb. Sp, and Ct. Th.

**Table 1:** Summary of the characteristics of experimental subjects

Study	Year	Experimental subject	Model	Sex	Age	Weight
Chen 1	2019	Mice	OVX-induced osteoporosis model	Female	–	–
Chen 2	2019	Mice	Senile osteoporosis model	Unspecified	–	–
Chen 3	2019	Mice	Disuse osteoporosis model (TS-induced osteoporosis model)	Unspecified	–	–
Gong	2020	SAMP8 mice	Senescence-accelerated model	Male	6 months old	–
Hu 1	2020	C57BL/6 mice	OVX-induced osteoporosis model	Female	8 weeks old	–
Hu 2	2020	C57BL/6 mice	Senile osteoporosis model	Male	16 months old	–
Hu 3	2020	C57BL/6 mice	Disuse osteoporosis model (TS-induced osteoporosis model)	Unspecified	3 months old	–
Huang	2021	SD rats	OVX-induced osteoporosis model	Female	10 weeks old	230–250 g
Li	2021	SD rats	OVX-induced osteoporosis model	Female	8 weeks old	294 ± 11 g
Lu	2020	C57BL/6J mice	–	Male	3 months old	–
Qiu	2021	SD rats	OVX-induced osteoporosis model	Female	12 weeks old	280–300 g
Wang	2022	C57BL/6 mice	OVX-induced osteoporosis model	Female	12 weeks old	28–30 g
Xiao	2021	C57BL/6J mice	Disuse osteoporosis model (HU-induced osteoporosis model)	Male	6 months old	–
Zhang	2021	SD rats	Diabetic osteoporosis model (STZ-induced diabetes)	Unspecified	8–10 weeks old	–
Zhang	2022	SD rats	Diabetic osteoporosis model (STZ-induced diabetes)	Male	8 weeks old	–

OVX, ovariectomy; TS, tail suspension; SAMP8, senescence-accelerated mouse prone eight; SD, Sprague Dawley; HU, hindlimb unloading; and STZ, streptozotocin

**Table 2:** Study characteristics of the included trials

Study	Year	Source of exosomes	Administration route	Treatment cycle	Frequency	Sample area
Chen 1	2019	USC (human)	Intravenous injection	2 months	Once a week	Femur
Chen 2	2019	USC (human)	Intravenous injection	3 months	Once a week	Femur
Chen 3	2019	USC (human)	Intravenous injection	3 weeks	Twice a week	Femur
Gong	2020	ESC (human)	Gavage	6 months	Once every other day	Femur
Hu 1	2020	UCMSC (human)	Intravenous injection	2 months	Once a week	Femur
Hu 2	2020	UCMSC (human)	Intravenous injection	3 months	Once a week	Femur
Hu 3	2020	UCMSC (human)	Intravenous injection	21 days	Twice a week	Femur
Huang	2021	BMSC (rat)	Intravenous injection	8 weeks	Once a week	Femur
Li	2021	BMSC (human)	Intravenous injection	28 days	Once a week	Tibia
Lu	2020	BMSC (rat)	Intravenous injection	2 months	Twice a week	Femur
Qiu	2021	BMSC (rat)	Intravenous injection	2 weeks	–	Femur
Wang	2022	BMSC (rat)	Periosteum injection	1 week	Twice a week	Femur
Xiao	2021	BMSC (rat)	Intravenous injection	4 weeks	Twice a week	Femur
Zhang	2021	ADSC (rat)	Intravenous injection	42 days	Once every other day	–
Zhang	2022	ADSC (rat)	Intravenous injection	12 weeks	Once every other day	Femur and tibia

USC, urine-derived stem cell; ESC, embryonic stem cell; UCMSC, umbilical cord-derived mesenchymal stromal cell; BMSC, bone marrow mesenchymal stem cell; and ADSC, adipose tissue-derived mesenchymal stem cell

### Methodology quality and risk of bias

**Fig. 3:**
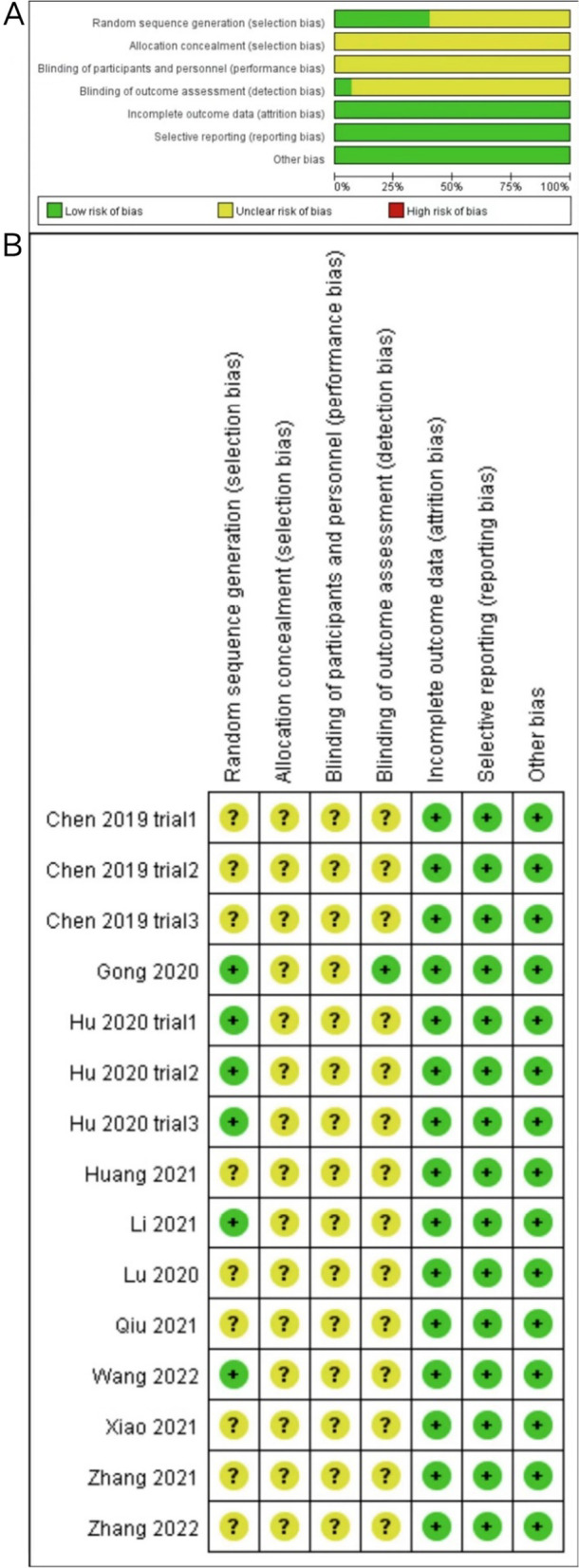
Risk of bias. **A** Graph showing bias risk. **B** Summary of bias risk

Six of the fifteen trials divided subjects into at least exosome treatment and control groups according to the principle of random assignment and were therefore judged to be at low risk of selection bias. However, the other nine trials did not mention their selection strategy. None of the studies revealed that the trials were conducted by assigning, concealing, and blinding caregivers and/or investigators. Only one trial reported blinding of the outcome assessment and was assigned as low risk in detection bias. Missing data, selectively reported data, or other biases in trials were not included in the meta-analysis. The methodological quality of the fifteen trials was reliable and acceptable (Fig. 3).

### Standard meta-analyses

#### BMD

**Fig. 4:**
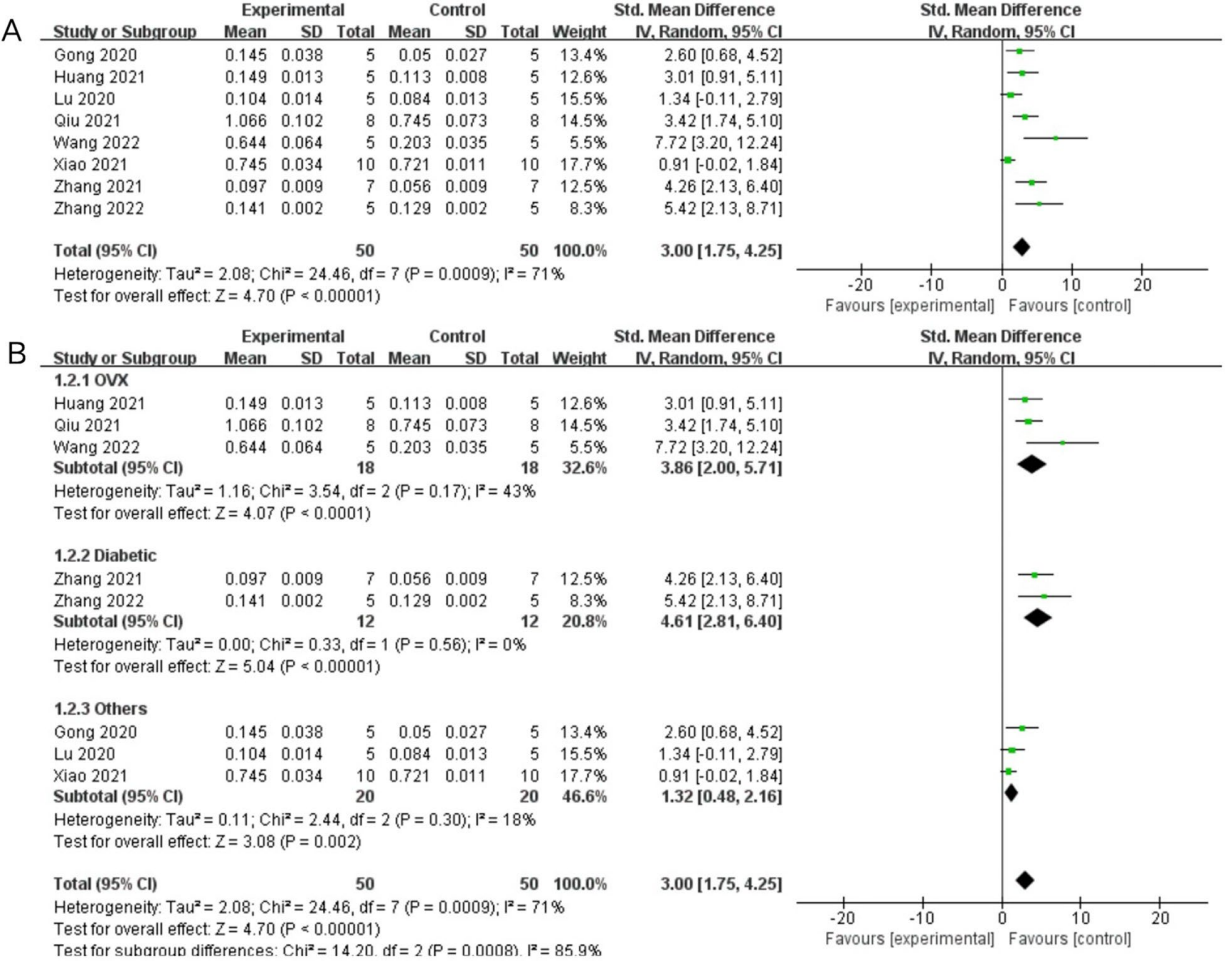
Forest plots depicting the comparison between the experimental and control groups: **A** Bone mineral density (BMD) and **B** subgroup analysis for BMD

**Fig. 5:**
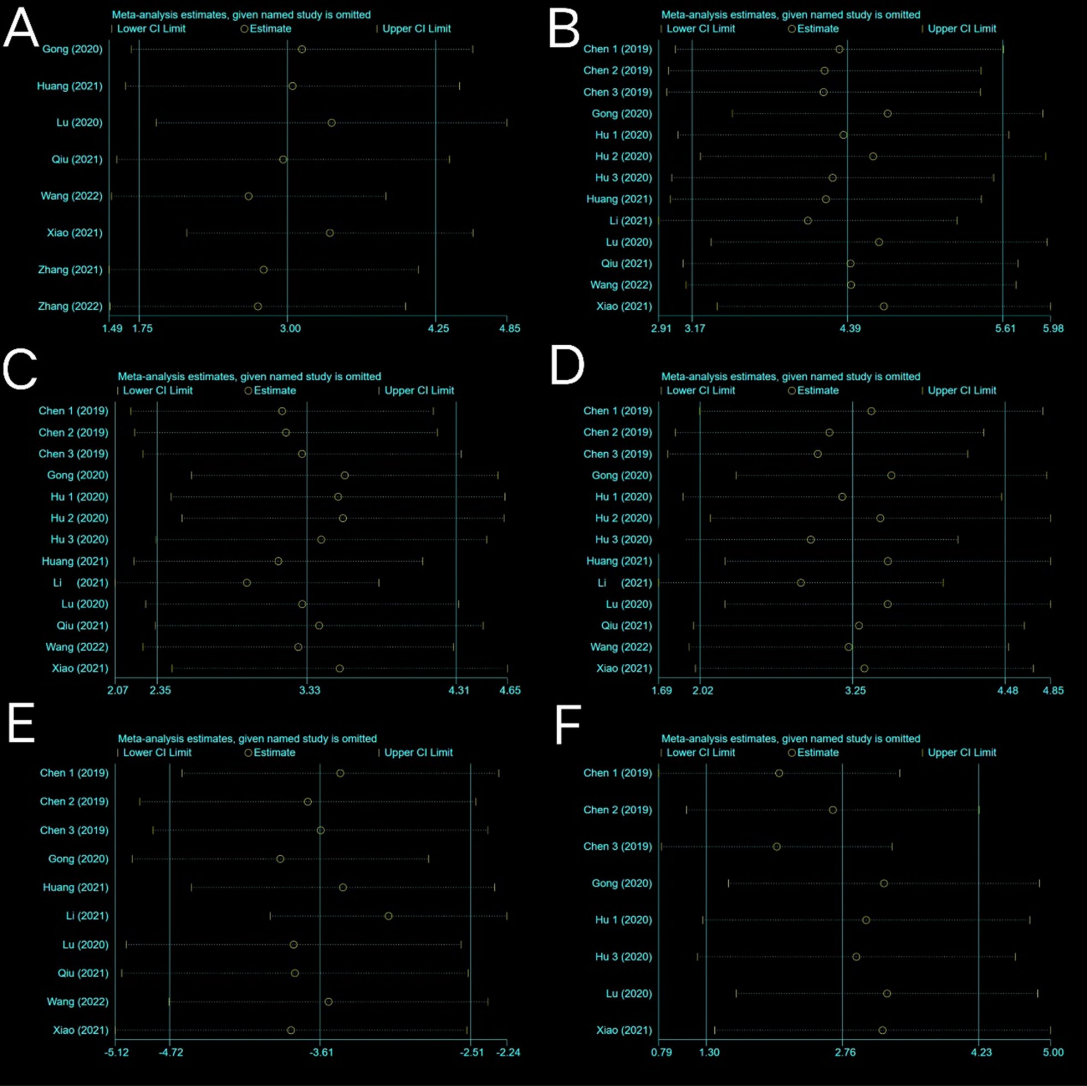
Sensitivity analysis. **A** Bone mineral density (BMD). **B** Bone volume fraction (trabecular bone volume/total volume, BV/TV). **C** Trabecular number (Tb. N). **D** Trabecular thickness (Tb. Th). **E** Trabecular separation/marrow thickness (Tb. Sp). **F** cortical thickness (Ct. Th)

Eight trials with a total of 50 subjects reported BMD in their experimental groups and control groups. The Q test and I^2^ test across studies exhibited significant heterogeneity (*P* = 0.0009 < 0.05, *I*^2^ = 71% > 50%). Results from the random-effects model were SMD = 3.00, 95% CI [1.75, 4.25], *P* < 0.00001 (Fig. 4A). Therefore, a subgroup analysis was performed to bring the heterogeneity below 50%. The trials were further divided into three subgroups including ovariectomy (OVX) and diabetes according to the different animal models. I^2^ was successfully reduced in each subgroup (OVX: *P* = 0.17 > 0.05, *I*^2^ = 43% < 50%; diabetic: *P* = 0.56 > 0.05, *I*^2^ = 0% < 50%; others: *P* = 0.30 > 0.05, *I*^2^ = 18% < 50%). Meanwhile, exosome therapy increased bone density, reducing the risk of bone loss and osteoporosis from the SMD results of the three subgroups (OVX: SMD = 3.86, 95% CI [2.00, 5.71], *P* < 0.0001; diabetic: SMD = 4.61, 95% CI [2.81, 6.4], *P* < 0.00001; others: SMD = 1.32, 95% CI [0.48, 2.16], *P* = 0.002) (Fig. 4B). The pooled size effect did not significantly change after the individual exclusion of trials in the sensitivity analysis (Fig. 5A). This indicated that the results were relatively robust and reliable.

#### BV/TV

**Fig. 6:**
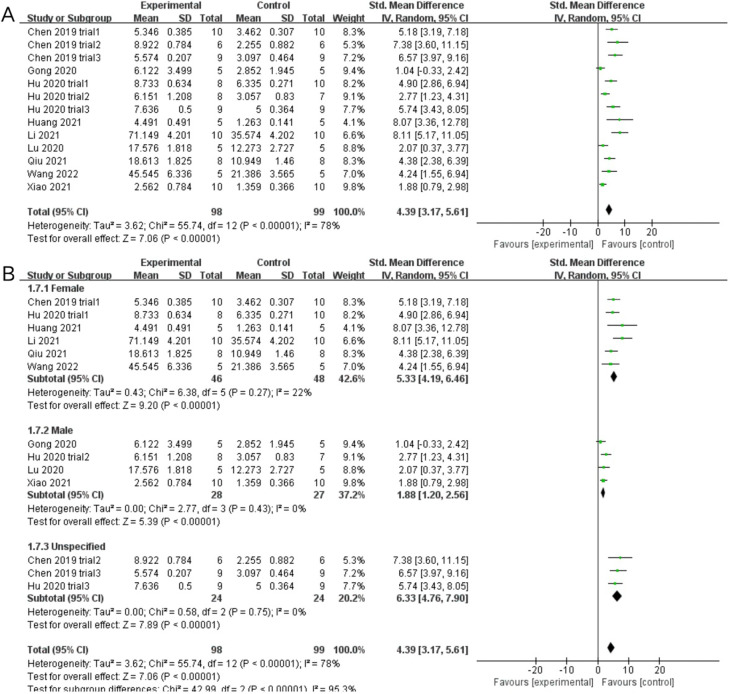
Forest plots depicting the comparison between the experimental and control groups: **A** BV/TV (bone volume/total volume) and **B** subgroup analysis for BV/TV

Nine studies including thirteen trials reported BV/TV data for exosome-treated and control groups. Subgroup analysis was conducted owing to significant heterogeneity of the included trials (*P* < 0.00001, *I*^2^ = 78%; SMD = 4.39, 95% CI [3.17, 5.61], *P* < 0.00001) (Fig. 6A). Each subgroup I^2^ was lower than 50% after dividing the trials into three subgroups based on the sex of the experimental animals (female: *P* = 0.27 > 0.05, *I*^2^ = 22% < 50%; male: *P* = 0.43 > 0.05, *I*^2^ = 0% < 50%; unspecified: *P* = 0.75 > 0.05, *I*^2^ = 0% < 50%). Meta-analysis results using the random-effects model were as follows: (female: SMD = 5.33, 95% CI [4.19, 6.46], *P* < 0.00001; male: SMD = 1.88, 95% CI [1.20, 2.56], *P* < 0.00001; unspecified: SMD = 6.33, 95% CI [4.76, 7.90], *P* < 0.00001). Exosome treatments significantly increased the BVF (compared with the placebo treatment) (Fig. 6B). This indicated that exosome therapy highly promoted bone anabolism rather than catabolism and improved bone metabolism. The results were considered to be robust as the pooled results did not significantly change during sensitivity analysis (Fig. 5B).

#### Tb. N

**Fig. 7:**
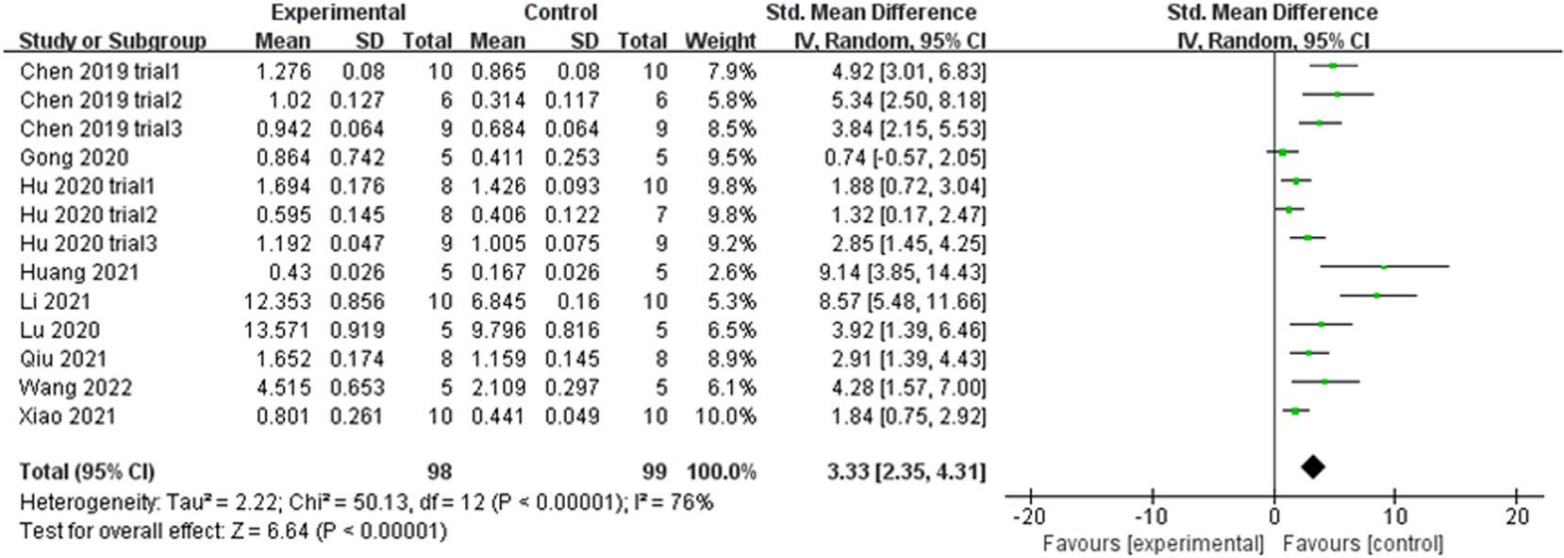
Forest plot of Tb. N. (trabecular number)

**Fig. 8:**
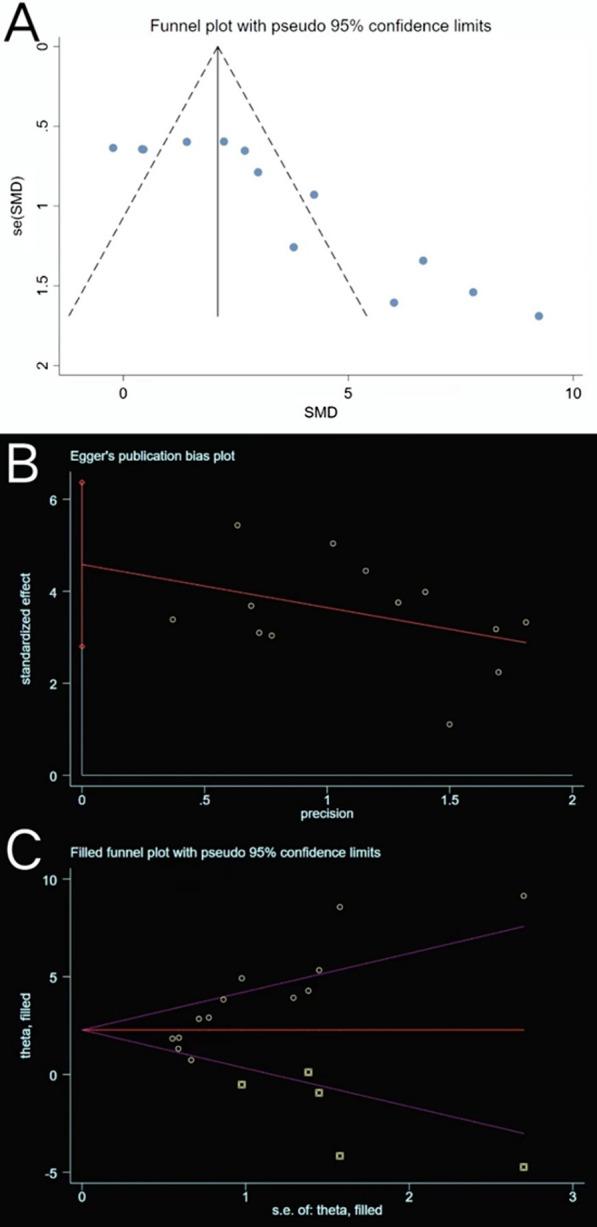
Plots of Tb. N. **A** Funnel plot with pseudo-95% confidence limits. **B** Egger’s publication bias plot. **C** Filled funnel plot with pseudo-95% confidence limits

Nine studies (including thirteen trials) reporting Tb. N data in experimental and control groups exhibited high heterogeneity (*P* < 0.00001, *I*^2^ = 76%) (Fig. 7). Subgroups were subsequently defined based on animal models, exosome sources, routes of administration, and sex of experimental subjects. However, subgroup heterogeneity did not improve. The funnel plot showed asymmetry when considering the effect of publication bias (Fig. 8A), and Egger’s test results of t = 5.65 and *P* = 0.000 < 0.05 proved the asymmetry. Therefore, the trim-and-fill method was used to evaluate the stabilisation of merged effect sizes (Fig. 8B). The results of both fixed and random effect model were reported; the random effect model was adopted (Q = 50.127, *P* = 0.000 < 0.05) and showed an estimate (Est) of 3.331 and 95% CI (2.348, 4.314). Five virtual studies were included (Fig. 8C), and the data were reanalysed. The results were Q = 89.816, *P* = 0.000 < 0.05, and the combined effect sizes were Est = 2.276 and 95% CI (1.235, 3.318) (Table 3). The difference between the two groups was significant (*P* = 0.000), despite publication bias. The contrary was not observed, showing the reliability of the meta-analysis. Moreover, no trial data showed small sample study effects according to sensitivity analysis, implying that the meta-analysis results were reliable (Fig. 5C). The analysis of Tb. N outcome indicator data with a random effect model showed an SMD of 3.33 (95% CI [2.35, 4.31]), which was considered statistically significant (test for overall effect: *P* < 0.00001). However, the result needs to be inferred with caution owing to the large heterogeneity. Despite this, all 13 trials showed that Tb. N increased in the experimental groups, indicating that exosome therapy ameliorated the impaired ability of bone anabolism induced in response to bone loss.

**Table 3:** Process of the trim-and-fill method for Tb. N (filled meta-analysis)

Method	Pooled Est	95% CI		Asymptotic		No. of studies
		Lower	Upper	z value	p value	
Fixed	2.098	1.680	2.515	9.838	0.000	18
Random	2.276	1.235	3.318	4.284	0.000	

Test for heterogeneity: *Q* = 89.816 on 17 degrees of freedom (*P* = 0.000). Moment-based estimate of between studies variance = 3.646

#### Tb. Th

**Fig. 9:**
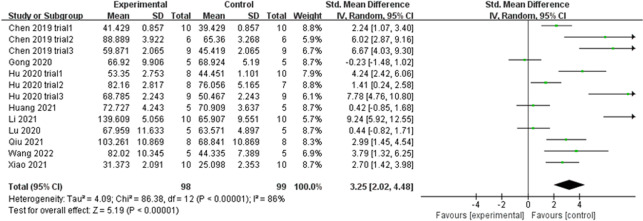
Forest plot of Tb. Th

**Fig. 10:**
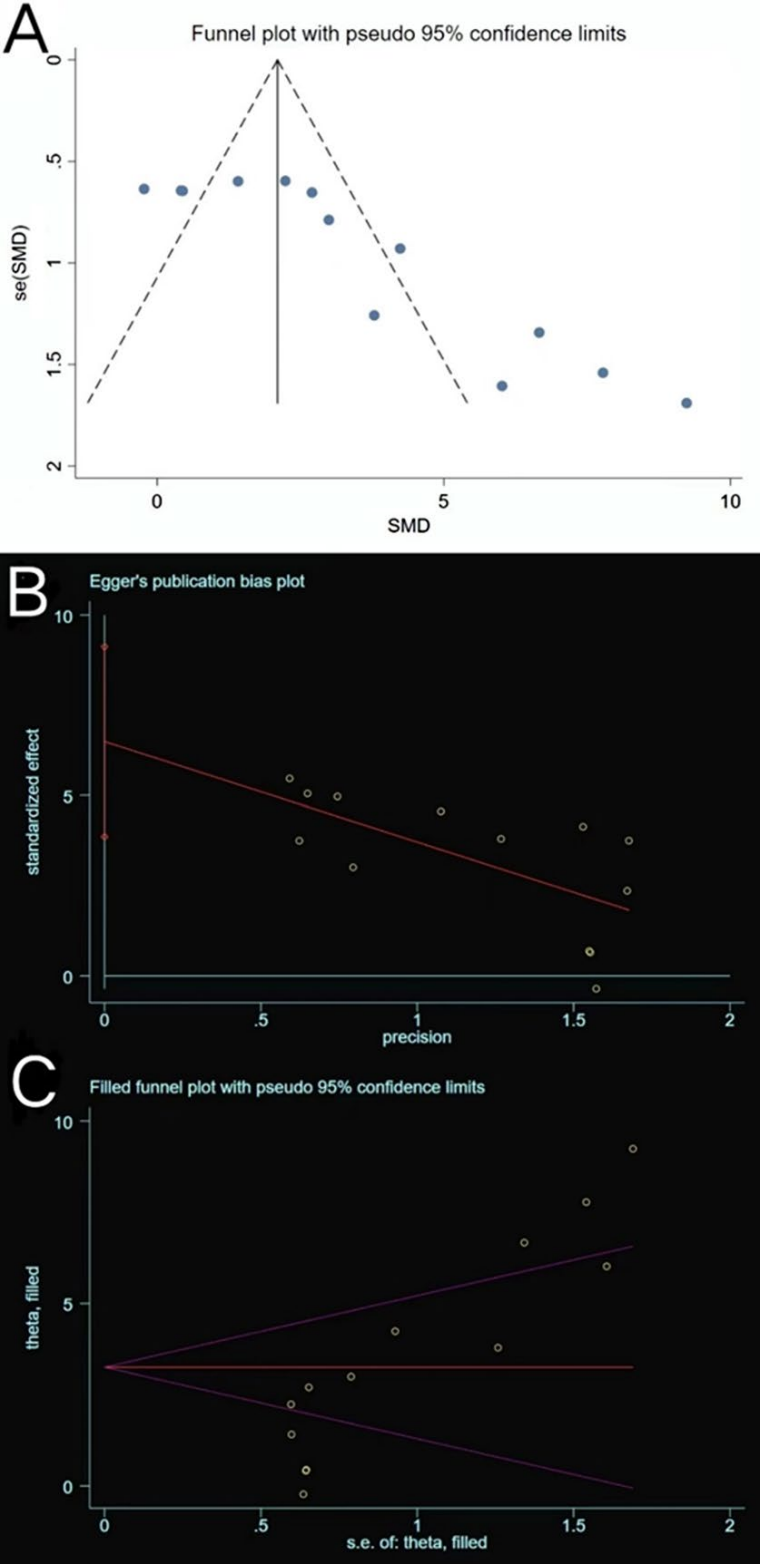
Plots of Tb. Th. **A** Funnel plot with pseudo-95% confidence limits. **B** Egger’s publication bias plot. **C** Filled funnel plot with pseudo-95% confidence limits

Similarly, Tb. Th extracted from 13 trials exhibited large heterogeneity (*P* < 0.00001, *I*^2^ = 86% > 50%) (Fig. 9). The heterogeneity did not decrease following a subgroup analysis based on the animal model, exosome source, route of administration, and sex of experimental subjects. The funnel plot (Fig. 10A) was asymmetric, and Egger’s test results (*P* = 0.000 < 0.05) (Fig. 10B) confirmed the publication bias. The trim-and-fill method indicated that no trimming was performed (Fig. 10C), and the data remained unchanged (Table 4). Sensitivity analysis established the rationality of the result (Fig. 5D). Therefore, the merged effect size of outcome indicator Tb. Th (SMD = 3.25, 95% CI [2.02, 4.48]) analysed with the random effect model was considered significant (*P* < 0.00001). The high sample heterogeneity indicates that the results should be inferred with caution; however, almost all sets of values (12 out of 13 trials) increased. This illustrates that the degree of bone anabolism was higher than that of bone catabolism in the exosome treatment groups (compared with placebo groups).

**Table 4:** Process of the trim-and-fill method for Tb. Th (filled meta-analysis)

Method	Pooled Est	95% CI		Asymptotic		No. of studies
		Lower	Upper	*z* value	*P* value	
Fixed	2.099	1.664	2.533	9.468	0.000	13
Random	3.253	2.025	4.481	5.191	0.000	

Test for heterogeneity: *Q* = 86.380 on 12 degrees of freedom (*P* = 0.000). Moment-based estimate of between studies variance = 4.089

#### Tb. Sp

**Fig. 11:**
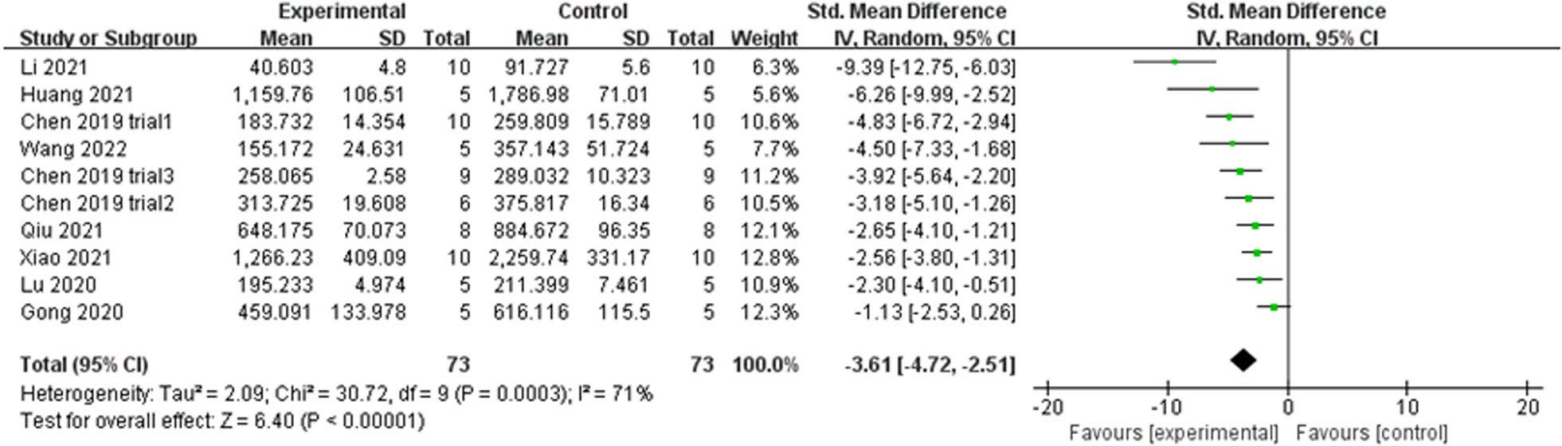
Forest plot of Tb. Sp

**Fig. 12:**
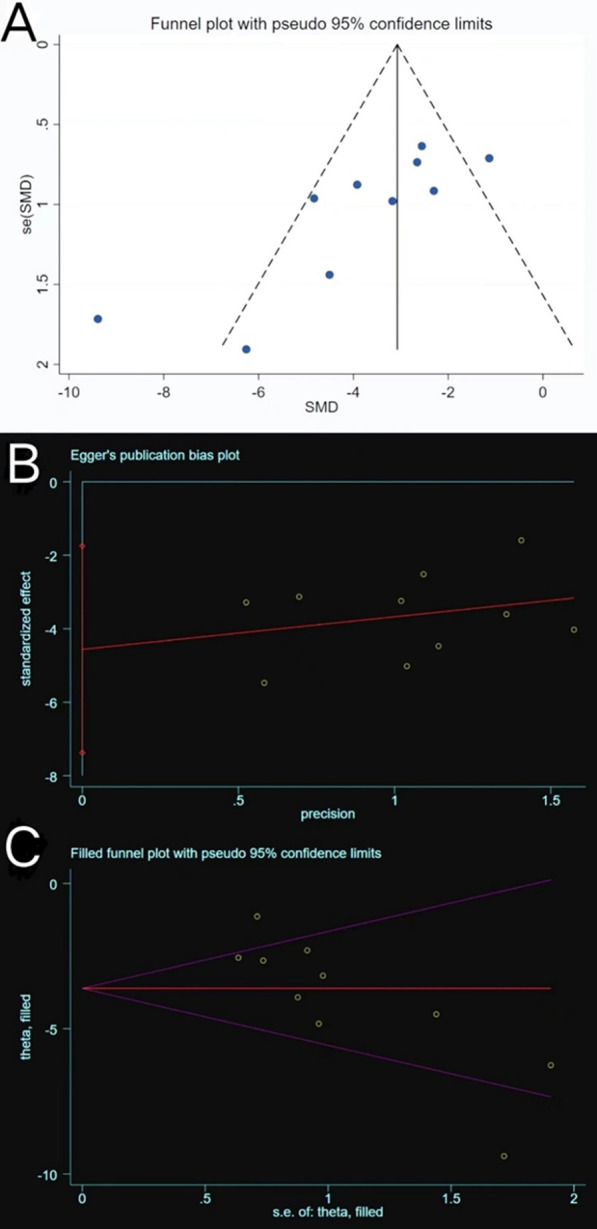
Plots of Tb. Sp. **A** Funnel plot with pseudo-95% confidence limits. **B** Egger’s publication bias plot. **C** Filled funnel plot with pseudo-95% confidence limits

Tb. Sp was reported in 10 of the included trials. High heterogeneity was observed (*P* = 0.0003 < 0.05, *I*^2^ = 71% > 50%) (Fig. 11), a phenomenon that was not ameliorated following subgroup analyses based on different study characteristics. The funnel plot revealed asymmetry (Fig. 12A), and Egger’s test (*t* = − 3.73, *P* = 0.006 < 0.05) (Fig. 12B) confirmed publication bias. The trim-and-fill method (Fig. 12C) indicated that no trimming was performed, and the data were unchanged (Table 5). This showed that the combined results were robust, and that the publication bias of the included studies was small and acceptable. Sensitivity analysis reflected the reliability of the results (Fig. 5E). All 10 trials generally reported a decline in Tb. Sp, and meta-analysis using the random effect model (SMD = − 3.61, 95% CI [− 4.72, − 2.51]) revealed that exosome therapy decreased the mean width of the medullary cavity between the bone trabeculae, thereby improving bone microstructure (Fig. 11).

**Table 5:** Process of the trim-and-fill method for Tb. Sp (filled meta-analysis)

Method	Pooled Est	95% CI		Asymptotic		No. of studies
		Lower	Upper	*z* value	*P* value	
Fixed	− 3.074	− 3.640	− 2.509	− 10.655	0.000	10
Random	− 3.613	− 4.720	− 2.506	− 6.396	0.000	

Test for heterogeneity: *Q* = 86.380 on 12 degrees of freedom (*P* = 0.000). Moment-based estimate of between studies variance = 4.089

#### Ct. Th

**Fig. 13:**
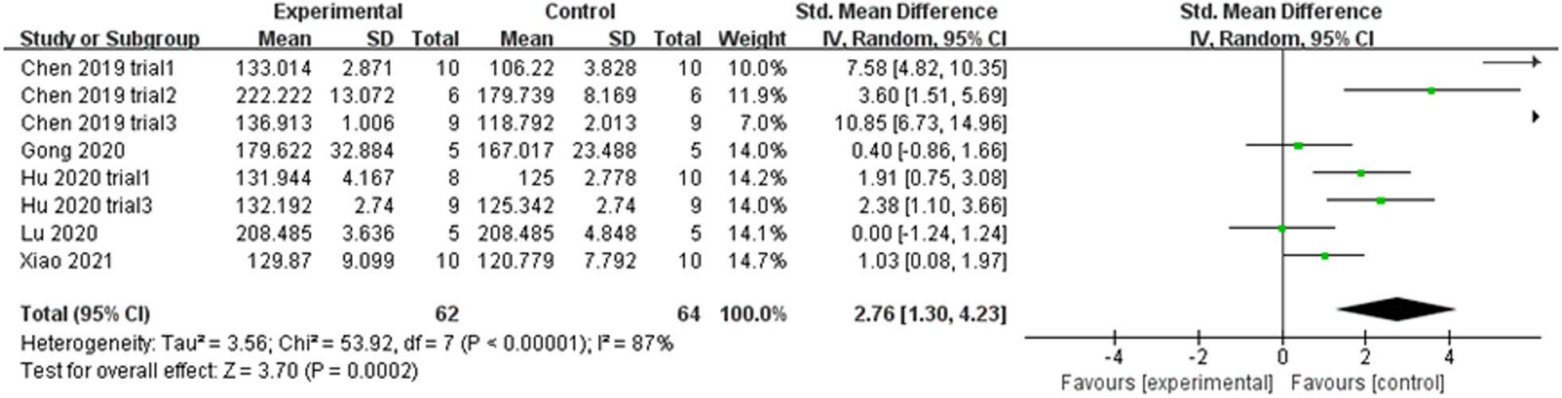
Forest plot of Ct. Th

A total of eight trials reported the value of Ct. Th. Data sets below 10 were not analysed using the funnel plot and Egger’s test owing to low reliability of the results. The sensitivity analysis results were stable (Fig. 5F). A random effect model was used in the meta-analysis (*P* < 0.00001, *I*^2^ = 87% > 50%), and there was a significant difference (*P* = 0.0002) in Ct. Th between the exosome treatment groups and placebo groups based on pooled analysis results (SMD = 2.76, 95% CI [1.30, 4.23]) (Fig. 13). Meanwhile, seven out of eight trials reported a significant increase in Ct. Th following exosome treatment.

Overall, exosomes can significantly increase the cortical thickness of the trabecular bone and enhance bone strength compared with the placebo treatment.

## Discussion

This meta-analysis investigated the efficacy of stem cell-derived exosomes in the treatment of osteoporosis. Stem cell-derived exosome therapy significantly improves bone repair and bone regeneration in osteoporosis (compared to placebo treatment) based on the overall results of all six bone-related indicators (BMD, BV/TV, Tb. N, Tb. Th, Tb. Sp, and Ct. Th).

**Fig. 14:**
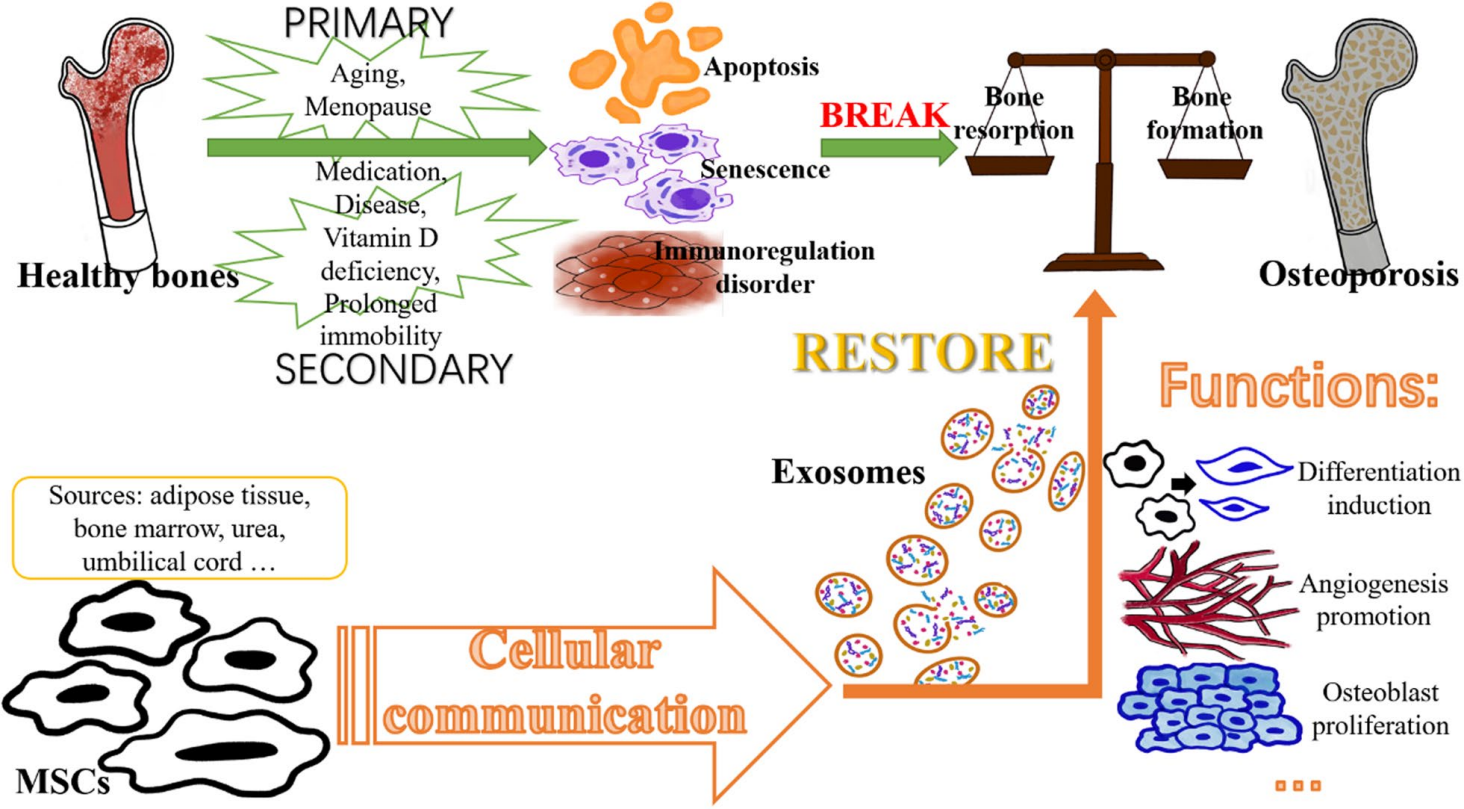
Summary graph illustrating the pathogenesis of osteoporosis and mechanism of exosome formation and release. MSCs, mesenchymal stem cells

Osteoporosis is a metabolic bone disease with complex aetiology that is primarily classified into two categories: primary osteoporosis and secondary osteoporosis. The former is prevalent in older men and postmenopausal women over the age of 50 years, whereas the latter is typically caused by adverse drug use, such as glucocorticoids. Diseases such as hyperthyroidism or other conditions (such as vitamin D deficiency and prolonged immobility) are also contributing factors [5, 36–38]. These pathogenic factors induce imbalance of bone homeostasis and disrupt cellular communication and related signalling factors and pathways, leading to enhanced inflammatory responses, MSC senescence, immune regulation disorders, osteocyte apoptosis, inhibited osteoblast differentiation, and an imbalance between bone resorption and formation [13, 39, 40]. Its negative effects may include increased adipogenesis, decreased bone density and strength, and destruction of bone tissue microarchitecture (Fig. 14). Trials using different animal models were included in the meta-analysis.

Exosomes are derived from stem cells and both have similar functions, including inhibiting inflammation and promoting angiogenesis. However, safety issues associated with the use of stem cells, including the risk of carcinogenesis and thrombosis along with low transplant efficiency (low homing ability of stem cells and high apoptosis rate), as well as concerns regarding the ethics (source of cell donation) of stem cell transplants, are unresolved [39, 41, 42]. Exosomes do not have these issues and have a wider range of sources and additional functions, such as repairing impaired MSCs and inducing osteogenic differentiation. In addition, exosomes are not immunogenic because they lack MHCI and MHCII proteins [39, 41]. Exosomes, or extracellular vesicles, play a key role in bone repair through cellular communication, and MSCs function through paracrine mechanisms [13, 42, 43]. Various nucleic acids (such as miRNAs, lncRNAs, and piRNAs), proteins, lipids, and other active molecules in exosomes stimulate bone repair via differentiation induction, osteoblast proliferation, apoptosis inhibition, angiogenesis promotion, and immune regulation [13, 44] (Fig. 14).

In general, existing osteoporosis treatment regimens have limitations [2]. For example, the incidence of atypical fractures in clinical practice drastically increases with prolonged bisphosphonate use in osteoporotic patients after the first three-years of treatment [45]. Long-term continuous hormone replacement therapy for postmenopausal osteoporosis may cause serious adverse effects, including breast cancer, endometrial hyperplasia, and venous thromboembolism [46–48]. Sudden cessation of denosumab leads to a rebound phenomenon, while long-term calcitonin use increases the risk of cancer [48]. In contrast, treatment of osteoporosis with various aetiologies using exosomes has high efficacy, and currently, there are no reported side effects. The increase in Tb. N and Tb. Th and decline in Tb. Sp observed in this meta-analysis revealed the improvement in spatial morphology and structure of bone trabecula. The increase in bone strength in cortical bones was well demonstrated by the increase in Ct. Th, and BMD subgroup analysis of animal models (Fig. 4B) confirmed the efficacy of exosomes in treating osteoporosis. The different results in the subgroups suggest that exosome efficacy in the context of osteoporosis depends on different stimuli. The mechanism may be related to the upregulation or downregulation of active components, particularly microRNAs. Similarly, it was speculated that the subject’s sex may also affect the exosome treatment efficacy based on the analysis of the sex-based BV/TV subgroup.

Subgroup analyses of Tb. N (Additional file 2: Fig. S1), Tb. Th (Additional file 2: Fig. S2), Tb. Sp (Additional file 2: Fig. S3), and Ct. Th (Additional file 2: Fig. S4) were separately performed based on animal model, sex, exosome source, and administration route. Despite the high heterogeneity, we hypothesised that the efficacy of exosome therapy may vary with these influencing factors, and that the variation likely results from different bioactive substances and their specific signal transduction pathways. However, this hypothesis needs to be supported by more well-designed studies with high-quality data to increase data homogeneity and reliability. Similarly, many factors related to exosome efficacy were neither analysed nor discussed owing to insufficient experimental quantity, limited sample size, and inconsistent study design (injection volume, injection frequency, and treatment cycle).

In addition, considering the differences between ESCs and MSCs, we reanalysed the data of the included six outcome assessment indices after rejecting the study on ESC-derived exosomes. Compared with the results before rejecting said study, despite minor data growth and reduction in pooled analysis results and heterogeneity, there were still significant differences in all observed indicators, which showed the therapeutical effect of exosomes derived from MSC in rat models of osteoporosis (Additional file 2: Fig. S5). The only study that focused on ESC-derived exosomes also came to a similar conclusion that this kind of exosome has certain therapeutic benefits. Moreover, it is difficult to determine the effect differences between exosomes derived from different stem cells as research on this subject is inadequate at present.

This meta-analysis has certain limitations. Studies conducted in other countries typically used exosomes from sources unqualified for this review, or exosomes modulated by genetic manipulation, which also disqualified them for the purposes of this review; consequently, all studies included in this review were conducted in China, which inevitably introduced a level of bias. Therefore, constant attention should be paid to related studies in order to update the analysis in the future, reducing the bias of the results. Another limitation of this article is that the quality of the included studies may vary owing to the lack of detailed methodological records. Furthermore, the high heterogeneity caused by injection dosage and frequency, treatment cycle, and other factors needs to be considered and improved in some indices. Standardised experimental conditions are required to ensure homogeneity. Additionally, the metric indicators were simplistic and only reflected the changes in bone mass and strength. Finally, all the trials were conducted in animal models; therefore, translating the results into clinical studies requires further investigation.

## Conclusions

This meta-analysis compared the efficacy of exosomes derived from stem cells in the context of osteoporosis (compared with a placebo) using six bone-related indicators. The overall results demonstrated that exosome therapy has a beneficial effect in treating osteoporosis in murine models. However, the potential usefulness of exosomes to treat osteoporosis in humans remains to be explored in larger, more biologically relevant animal models, and further investigation is required to understand the possible mechanisms of action. In addition, there needs to be a consensus in the scientific community regarding a research plan and technical route to improve data homogeneity for studies in this field. This will ensure reliable results. Further studies should be conducted to confirm the optimal therapeutic conditions for exosome therapy, including dosage, concentration, treatment cycle, and other aspects. More research using extracellular vesicles is required before exosome therapy for osteoporosis for successful translation to clinical trials.

## Supplementary Information

**Additional file 1**. Search strategy.

**Additional file 2**. Subgroup analysis and forest plots.

## Data Availability

All data generated or analysed during this study are included in this published article and its supplementary information files.

## Abbreviations

ADSC: Adipose tissue-derived mesenchymal stem cell
BMD: Bone mineral density
BMSC: Bone marrow-derived mesenchymal stem cell
CI: Confidence interval
Ct. Th: Cortical thickness
ESC: Embryonic stem cell
Est: Estimate
hESC: Human embryonic stem cell
HU: Hindlimb unloading
hucMSC: Human umbilical cord
IV: Intravenous
MSC: Mesenchymal stromal cell
OVX: Ovariectomy
SAMP8: Senescence-accelerated mouse prone eight
SD: Standard deviation
SEM: Standard error of the mean
SMD: Standardised mean difference
STZ: Streptozotocin
Tb.BV/TV: Trabecular bone volume/tissue volume fraction or bone volume fraction (BVF)
Tb. N: Trabecular number
Tb. Sp: Trabecular separation
Tb. Th: Trabecular thickness
TS: Tail suspension
USC: Urine-derived stem cell
VCF: Vertebral compression fracture
WMD: Weighted mean difference
