# Supplementary material for: Osteoporosis treatment using stem cell-derived exosomes: a systematic review and meta-analysis of preclinical studies
Source: Stem Cell Res Ther. 2023 Apr 11;14:72. doi: 10.1186/s13287-023-03317-4 (PMC10088147; doi:10.1186/s13287-023-03317-4)
Supplement: Supplementary file 1 — Additional file 1. Search strategy. [file 13287_2023_3317_MOESM1_ESM.docx]

**Table 1.** Search strategy

| **DATABASE** | **MeSH words AND Free words** | **Results** |
| --- | --- | --- |
| PubMed | (((((((((((Extracellular Vesicle) OR (Vesicle, Extracellular)) OR (Vesicles, Extracellular)) OR (Exovesicles)) OR (Exovesicle)) OR (Apoptotic Bodies)) OR (Apoptotic Body)) OR (Bodies, Apoptotic)) OR (Body, Apoptotic)) OR ("Extracellular Vesicles"[Mesh])) OR ("Exosomes"[Mesh])) AND (((((((((((((((((((((Osteoporoses) OR (Osteoporosis, Post-Traumatic)) OR (Osteoporosis, Post Traumatic)) OR (Post-Traumatic Osteoporoses)) OR (Post-Traumatic Osteoporosis)) OR (Osteoporosis, Senile)) OR (Osteoporoses, Senile)) OR (Senile Osteoporoses)) OR (Osteoporosis, Involutional)) OR (Senile Osteoporosis)) OR (Osteoporosis, Age-Related)) OR (Osteoporosis, Age Related)) OR (Bone Loss, Age-Related)) OR (Age-Related Bone Loss)) OR (Age-Related Bone Losses)) OR (Bone Loss, Age Related)) OR (Bone Losses, Age-Related)) OR (Age-Related Osteoporosis)) OR (Age Related Osteoporosis)) OR (Age-Related Osteoporoses)) OR (Osteoporoses, Age-Related)) | 152 |
| Embase | (('osteoporosis'/exp) OR ('osteoporoses') OR ('osteoporosis, post-traumatic') OR ('osteoporosis, post traumatic') OR ('post-traumatic osteoporoses') OR ('post-traumatic osteoporosis') OR ('osteoporosis, senile') OR ('osteoporoses, senile') OR ('senile osteoporoses') OR ('osteoporosis, involutional') OR ('senile osteoporosis') OR ('osteoporosis, age-related') OR ('osteoporosis, age related') OR ('bone loss, age-related') OR ('age-related bone loss') OR ('age-related bone losses') OR ('bone loss, age related') OR ('bone losses, age-related') OR ('age-related osteoporosis') OR ('age related osteoporosis') OR ('age-related osteoporoses') OR ('osteoporoses, age-related')) AND (('exosome'/exp) OR ('extracellular vesicles') OR ('extracellular vesicle') OR ('vesicle, extracellular') OR ('vesicles, extracellular') OR ('exovesicles') OR ('exovesicle') OR ('apoptotic bodies') OR ('apoptotic body') OR ('bodies, apoptotic') OR ('body, apoptotic')) | 198 |
| Cochrane Library | (((((((((((((((((((((MeSH descriptor:[Osteoporosis] explode all trees) OR (Osteoporoses)) OR(Osteoporosis, Post-Traumatic)) OR (Osteoporosis, Post Traumatic)) OR (Post-Traumatic Osteoporoses)) OR (Osteoporosis, Senile)) OR (Osteoporoses, Senile)) OR (Senile Osteoporoses)) OR (Osteoporosis, Involutional)) OR (Senile Osteoporosis)) OR (Osteoporosis, Age-Related)) OR (Osteoporosis, Age Related)) OR (Bone Loss, Age-Related)) OR (Age-Related Bone Loss)) OR (Age-Related Bone Losses)) OR (Bone Loss, Age Related)) OR (Bone Losses, Age-Related)) OR (Age-Related Osteoporosis)) OR (Age Related Osteoporosis)) OR (Age-Related Osteoporoses)) OR (Osteoporoses, Age-Related)) AND ((((((((((MeSH descriptor:[Extracellular Vesicles] explode all trees) OR (Extracellular Vesicle) OR (Vesicle, Extracellular)) OR (Vesicles, Extracellular)) OR (Exovesicles)) OR (Exovesicle)) OR (Apoptotic Bodies)) OR (Apoptotic Body)) OR (Bodies, Apoptotic)) OR (Body, Apoptotic)) OR (MeSH descriptor:[Exosomes] explode all trees) | 16 |
