# Supplementary material for: Osteoporosis treatment using stem cell-derived exosomes: a systematic review and meta-analysis of preclinical studies
Source: Stem Cell Res Ther. 2023 Apr 11;14:72. doi: 10.1186/s13287-023-03317-4 (PMC10088147; doi:10.1186/s13287-023-03317-4)
Supplement: Supplementary file 2 — Additional file 2. Subgroup analysis and forest plots. [file 13287_2023_3317_MOESM2_ESM.docx]

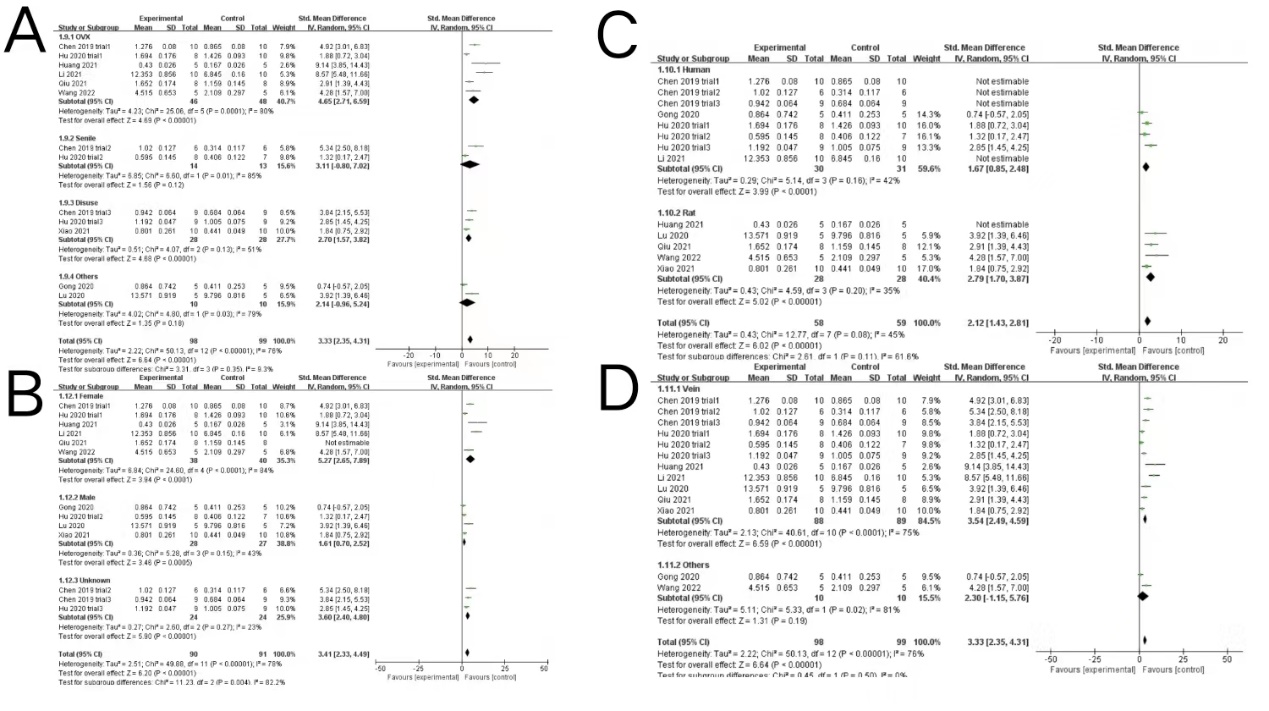


**Figure 1**. Subgroup analysis for Tb. N based on the animal model **(A)**, animal sex **(B)**, exosome source **(C)**, and administration route **(D)**.


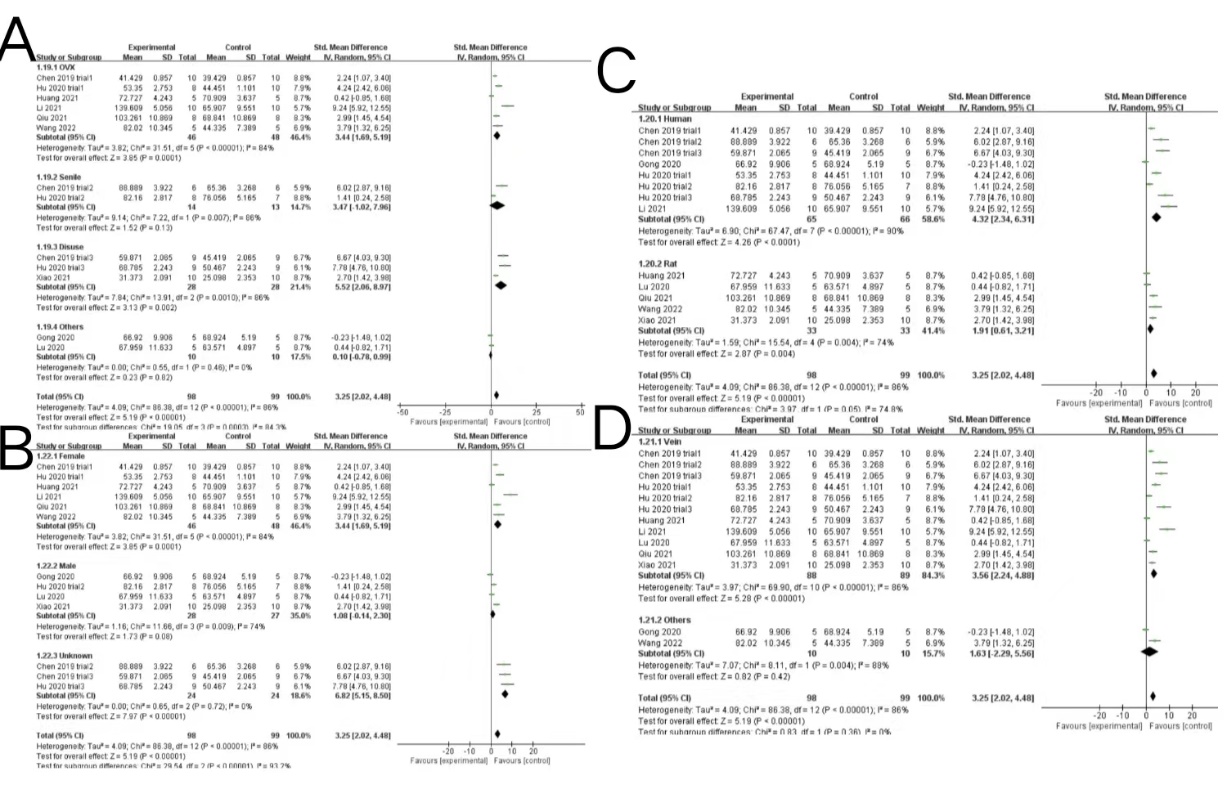


**Figure 2**. Subgroup analysis for Tb. Th based on the animal model **(A)**, animal sex **(B)**, exosome source **(C)**, and administration route **(D)**.


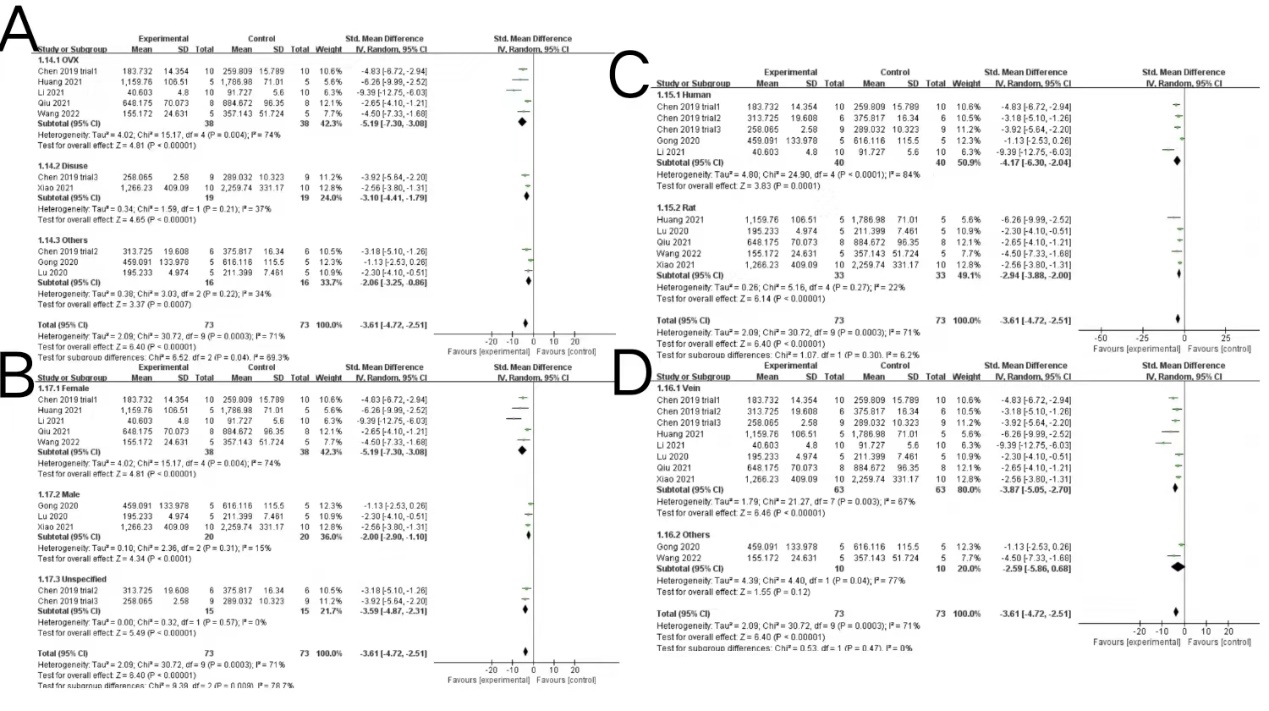


**Figure 3.** Subgroup analysis for Tb. Sp based on the animal model **(A)**, animal sex **(B)**, exosome source **(C)**, and administration route **(D)**.


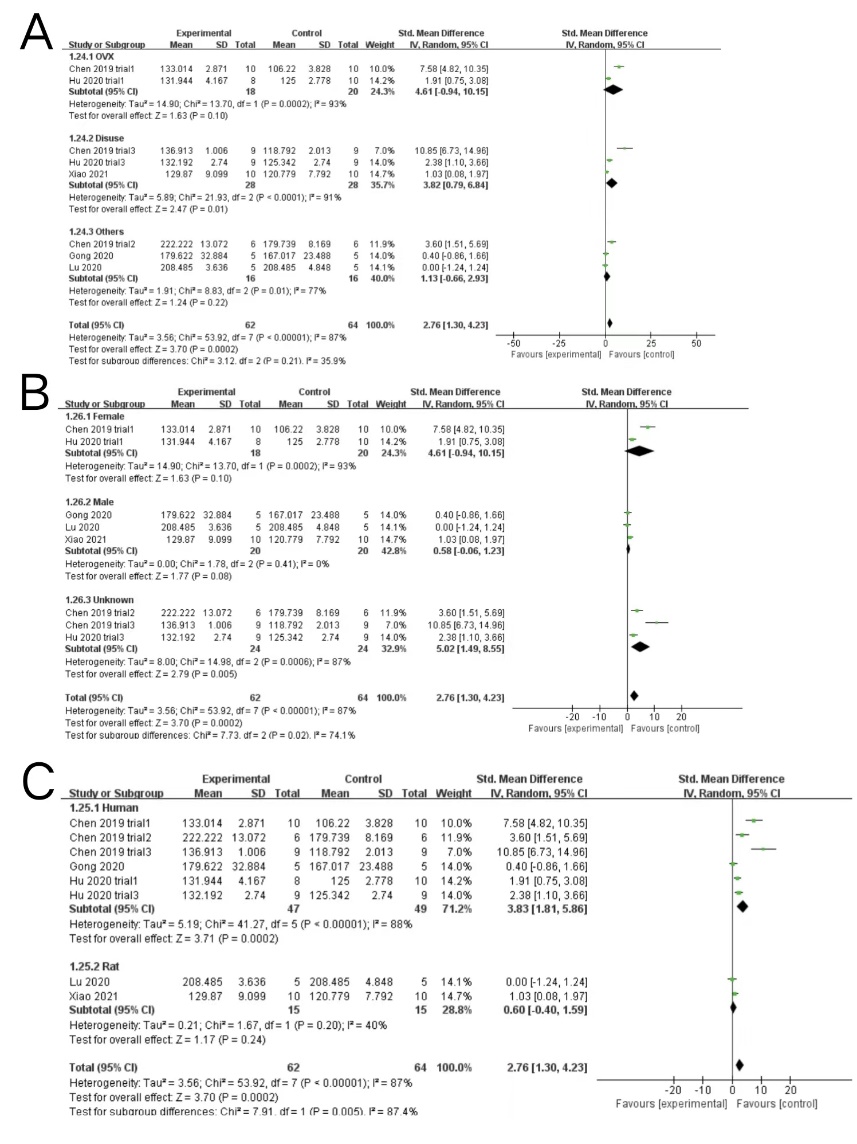


**Figure 4**. Subgroup analysis for Ct. Th based on the animal model **(A)**, animal sex **(B)**, and exosome source **(C)**.


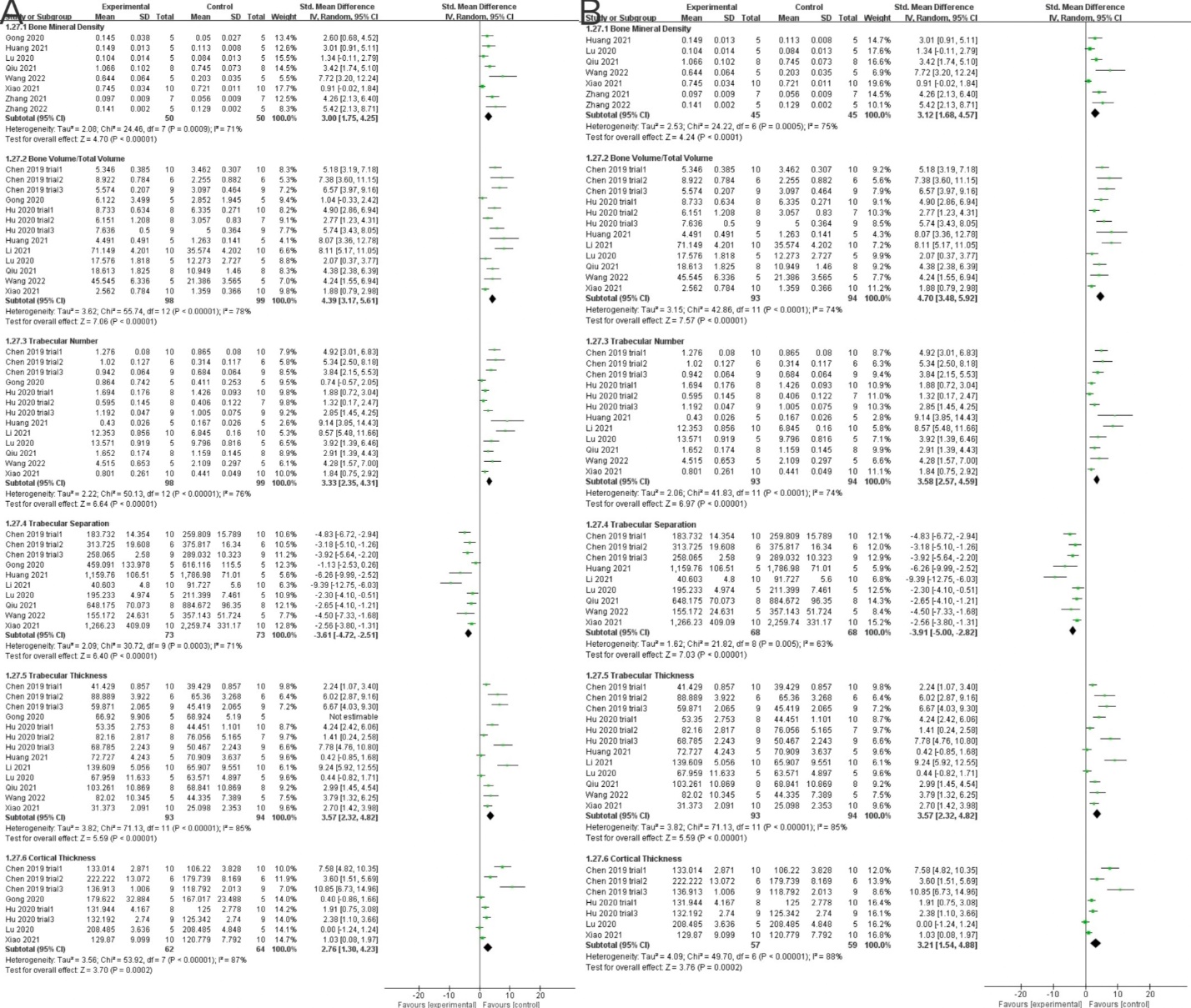


**Figure 5**. Forest plots of all six outcome assessment indices separately including 15 studies **(A)** and 14 studies **(B)**.
